# An Immediate and Long-Term Complication of COVID-19 May Be Type 2 Diabetes Mellitus: The Central Role of β-Cell Dysfunction, Apoptosis and Exploration of Possible Mechanisms

**DOI:** 10.3390/cells9112475

**Published:** 2020-11-13

**Authors:** Melvin R. Hayden

**Affiliations:** Departments of Internal Medicine, Endocrinology Diabetes and Metabolism, Diabetes and Cardiovascular Disease Center, University of Missouri-Columbia School of Medicine, Columbia, MO 65212, USA; mrh29pete@gmail.com; Tel.: +1-573-346-3019

**Keywords:** ACE2, amylin, β-cell apoptosis, islet, islet amyloid, fibrosis, metabolic syndrome, oxidative stress, renin–angiotensin–aldosterone-system, SARS-CoV-2

## Abstract

The novel coronavirus disease 2019 (COVID-19) caused by the severe acute respiratory syndrome coronavirus-2 (SARS-CoV-2) was declared a pandemic by the WHO on 19 March 2020. This pandemic is associated with markedly elevated blood glucose levels and a remarkable degree of insulin resistance, which suggests pancreatic islet β-cell dysfunction or apoptosis and insulin’s inability to dispose of glucose into cellular tissues. Diabetes is known to be one of the top pre-existing co-morbidities associated with the severity of COVID-19 along with hypertension, cardiocerebrovascular disease, advanced age, male gender, and recently obesity. This review focuses on how COVID-19 may be responsible for the accelerated development of type 2 diabetes mellitus (T2DM) as one of its acute and suspected long-term complications. These observations implicate an active role of metabolic syndrome, systemic and tissue islet renin–angiotensin–aldosterone system, redox stress, inflammation, islet fibrosis, amyloid deposition along with β-cell dysfunction and apoptosis in those who develop T2DM. Utilizing light and electron microscopy in preclinical rodent models and human islets may help to better understand how COVID-19 accelerates islet and β-cell injury and remodeling to result in the long-term complications of T2DM.

## 1. Introduction

The intersection of metabolic syndrome (MetS), type 2 diabetes mellitus (T2DM) and the severe acute respiratory syndrome coronavirus-2 (SARS-CoV-2) virus responsible for coronavirus disease (COVID-19) may have not only immediate but also long-term complications. Indeed, COVID-19 may have a dynamic impact on the long-term complications regarding the development of T2DM and/or progression of orally controlled T2DM to an insulin-dependent type of T2DM.

**Figure 1:**
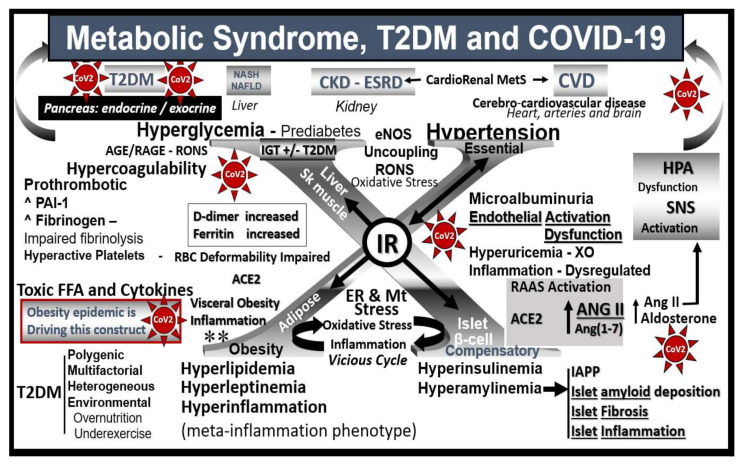
Metabolic syndrome, T2DM and COVID-19 are multisystem diseases. This image illustrates how metabolic syndrome (MetS)/type 2 diabetes mellitus (T2DM) and coronavirus disease 2019 (COVID-19) are two multisystem diseases that can have a tremendous interaction, with multiple crosstalk when they intersect. The central “X” in this figure honors Jerry Reaven who initially coined the term Syndrome X and championed the concept that resistance to insulin-mediated glucose disposal was a characteristic of patients with T2DM and cardiovascular disease (CVD), which was later termed MetS. There are four arms to this letter X and each arm has a designated condition to further illustrate the “H” phenomenon, representing a “hyper” state, i.e., hyperlipidemia, lower left; islet β-cell hyperinsulinemia/hyperamylinemia, lower right; hypertension, upper right; hyperglycemia, upper left. Note how insulin resistance (IR) is central to each of the four arms. While each arm is important, one can note that hyperinsulinemia and hyperamylinemia are of great importance to this review, in that this arm represents the hormonal secretion by the pancreatic β-cells that have the ACE2 on their outer surface that is necessary for SARS-CoV-2 (red spiked icon with CoV-2 labeling) to enter the cells. Further, ACE2 is present on the intra-islet microcirculation capillary endothelial cells/pericytes and the peri-islet capillaries. In addition to intra-islet amyloid deposition and fibrosis, there is also peri-islet amyloid and fibrosis, redox stress oxidative/nitrosative stress (RONS) and inflammation that are in a vicious cycle with one another. MetS and T2DM are known to be associated with the renin–angiotensin–aldosterone system (RAAS) within the islet and there exists the possibility that further activation of islet RAAS may be due to the diminished ACE2/Ang(1–7)/MasR as a result of viral virion binding and contribute to ongoing remodeling over time following the recovery from COVID-19. Additionally, due the overriding effect of Ang II excess due to ACE2 binding, there will be increased vasospasm and hypoxia to the islets that may compound the COVID-19 islet injury. Endothelial activation/dysfunction due MetS, T2DM and COVID-19 may be responsible for further islet damage. Importantly, there is the known cytokine storm that could initially play a damaging role to the islet and its contents with loss of β-cells. Further, cerebrocardiovascular disease (CVD) and chronic kidney disease (CKD) together comprise the brain–heart–kidney axis that is involved when there is vascular stiffness associated with MetS and T2DM. It is very important to note that only body mass index (BMI)/obesity (not morbid obesity) turned out to be independently associated with the primary outcome of need for ventilation and/or death at 7 days post-admission in the French CORONADO study and thus implicates obesity as a major predicting phenotype associated with the need for supportive ventilation and or death as obesity is also noted to be driving MetS (lower left-hand side of figure **) see reference 21. ACE = angiotensin-converting enzyme; ACE2 = angiotensin-converting enzyme 2; AGE = advanced glycation end products; ANGII/Ang II = angiotensin II; Ang(1–7) = angiotensin 1–7; CKD = chronic kidney disease; CVD = cerebrocardiovascular disease; D-dimer = a fibrin degradation product and is named after two D fragments of the fibrin protein joined by a crosslink upon fibrinolysis; eNOS = endothelial nitric oxide synthase; ER = endoplasmic reticulum; ESRD = end-stage renal disease; FFA = free fatty acids; HPA = hypothalamic–pituitary–adrenal axis; IAPP = islet amyloid polypeptide; MasR = MAS-related G protein-coupled receptor; Mt = mitochondria; NAFLD = non-alcoholic fatty liver disease; NASH = non-alcoholic steatohepatitis; PAI-1 = plasminogen activator-1; RAGE = receptor for AGE; RBC = red blood cell; RONS = reactive oxygen and nitrogen (nitrosative stress) species; SNS = sympathetic nervous system.

Data from the French CORONADO (SARS-CoV-2 and diabetes outcome) observational study (a nationwide French multicenter center (53) study in people with diabetes hospitalized for COVID-19 during March 2020) with a primary outcome end point of combined tracheal intubation for mechanical ventilation and/or death within 7 days of admission demonstrated the following: dyspnea (OR 2.10 [1.31, 3.35]), lymphopenia (OR 0.67 [0.50, 0.88]), increased C-reactive protein (OR 1.93 [1.43, 2.59]) and aspartate aminotransferase (AST) (OR 2.23 [1.70, 2.93]) levels, advanced age (OR 2.48 [1.74, 3.53]) and treated obstructive sleep apnea (OR 2.80 [1.46, 5.38]) were independent predictors of the primary outcome. Additionally, microvascular (OR 2.14 [1.16, 3.94]) and macrovascular complications (OR 2.54 [1.44, 4.50]) were also independently associated with the risk of death on hospitalized day 7 [1]. Interestingly, these authors were also able to conclude that body mass index (BMI), but not long-term glucose control, was positively and independently associated with tracheal intubation and/or death within 7 days. Notably, ACE2 staining expression in adipose tissue is known to be higher than pulmonary tissue and contribute to the pre-COVID-19 MetS and T2DM chronic low-grade inflammatory state once it becomes affected as an immediate stressor and could contribute to an ongoing source of chronic inflammation (meta-inflammation) in post-COVID-19 recovery and long-term complications [2,3,4]. These findings strongly suggest that obesity in addition to other co-morbidities plays an important role in COVID-19 just as it does in the development of MetS and T2DM (Figure 1).

**Figure 2:**
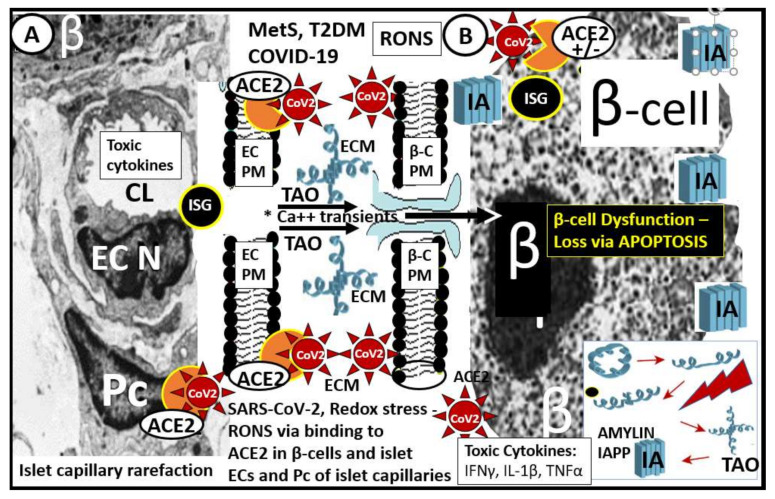
Pancreatic islet β-cells undergo a multiple hit injury with MetS/T2DM and COVID-19. This illustration depicts the close relationship of the islet capillaries and the β-cells with transmission electronic microscopic (TEM) images of the pancreatic islet capillary Panel (**A**) and the β-cell Panel (**B**) in those infected by SARS-CoV-2. Panel (**A**) depicts an islet capillary in close proximity to an islet β-cell (preclinical HIP rodent model) and notes that CoV-2 may bind not only to the endothelial cell ACE2 protein of capillary endothelial cells but may also bind to pericytes, since ACE2 has recently been found to bind to pericytes in the brain and myocardial capillary specimens especially if the EC glycocalyx barrier function has been damaged allowing SARS-CoV-2 to enter the subendothelial space. Panel (**B**) illustrates the small electron dense dots that are the insulin secretory granules (ISG) within the β-cell cytoplasm (preclinical HIP rodent model). Additionally, amylin undergoes unfolding and misfolding to form islet amyloid polypeptide (IAPP) (panel (**B**)—lower right hand) when exposed to toxic oxidative stress—reactive oxygen and nitrogen (nitrosative stress) species (RONS). The early more intermediate-sized toxic amyloid oligomers (TAO) of amylin have a propensity to form membrane permeant channels in the islet β-cell plasma membrane, which allow for the entrance of calcium transients to enter the β-cell and result in not only β-cell dysfunction but also β-cell loss via apoptosis see reference 63 and 64. There is approximately a 50% decrease in β-cell function of those with T2DM and a 40–50% loss of β-cells in individuals with impaired glucose tolerance or prediabetes. Therefore, one can deduce that if there is already this much β-cell loss why SARS-CoV-2 could significantly add to this loss via SARS-CoV-2 binding to the ACE2 on β-cells (+/−) with further β-cell dysfunction, apoptosis and possibly accelerate the natural history of T2DM. Note the (+/−) regarding the presence of the ACE2 enzyme receptor since there is currently some controversy regarding its presence in pancreatic islet β-cells; see Section 6 reference [70,71,72,73]. Notably, systemic toxic cytokines liberated from pulmonary tissues and systemic immune cells can also be related to pancreatic islet injury mechanisms. Additionally, in preclinical T2DM rodent models, there is intra-islet capillary rarefaction that may contribute to β-cell dysfunction and death see reference 66. ACE2 = angiotensin-converting enzyme 2 (orange color); β = β-cell; β-C = β-cell; Ca++ = calcium; CoV-2 = SARS-CoV-2 (spiked red icon); CL = capillary lumen; EC = endothelial cell; ECM = extracellular matrix; IA = islet amyloid; IAPP = islet amyloid polypeptide deposition (blue fibril icon); IFN-γ = interferon gamma; Il-1β = interleukin 1 beta; ISG = insulin secretory granules; MetS = metabolic syndrome; N = nucleus; Pc = pericyte; PM = plasma membrane; RONS = reactive oxygen and nitrogen (nitrosative stress) species; TAO = toxic intermediate-sized amyloid oligomers; TNFα = tumor necrosis alpha; T2DM = type 2 diabetes mellitus.

**Figure 3:**
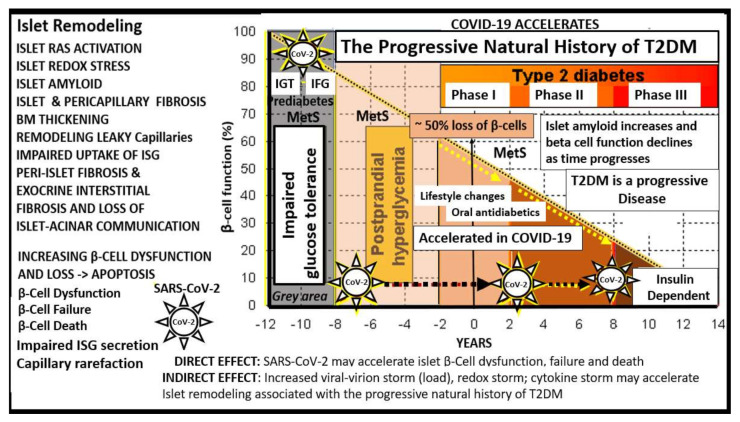
T2DM is a progressive disease with multiple stages and phases in humans. At the time of diagnosis of T2DM, patients may already have a 50% reduction in β-cell function. As one moves through the various stages/phases, there is a progressive loss of β-cell function, while concurrently there is increased deposition of amylin-derived islet amyloid and islet fibrosis (islet remodeling—isletopathy). When SARS-CoV-2 (spiked icon) binds to the ACE2 receptor of β-cells and vascular EC/pericyte, cells there will be a detrimental increase in the ratio of the ANG II–ACE2–MasR axis. COVID-19 is known to be associated with a systemic and even local cytokine storm in addition to a redox storm (excessive oxidative stress—reactive oxygen and nitrogen (nitrosative stress) species (RONS)) and could be possibly based on the viral virion (viral load) storm associated with viremia. Therefore, COVID-19 could accelerate the natural history of T2DM. This image is based on a classic graph by Lebovitz HE. Diabetes Rev. 1999, 7, 139–153 and Hayden MR et al. JOP. J. Pancreas (online) 2002, 3(5), 126–138. BM = basement membrane; CoV-2 = abbreviation of SARS-CoV-2; EC = endothelial cell; IFG = impaired fasting glucose; IGT = impaired glucose tolerance; ISG = insulin secretory granule; MetS = metabolic syndrome; T2DM = type 2 diabetes mellitus.

A better understanding of how the non-communicable T2DM/MetS global pandemic-like disease that existed long before the current highly communicable COVID-19 pandemic converge, intersect and interact is essential. Of equal importance is how their resultant multiple end-organ complications merit a better understanding of these two disparate syndromes/diseases (MetS and T2DM) and how COVID-19 may act to accelerate the natural history of T2DM (Figure 1, Figure 2 and Figure 3).

Importantly, the islet remodeling changes that exist prior to the infection with COVID-19 will have a marked impact on the sequela as manifested in the immediate- as well as the long-term complications of COVID-19, in that, COVID-19 will accelerate the natural history or phases from the prediabetes metabolic syndrome through phases I–III in T2DM of Figure 3. 

Indeed, the findings of hyperglycemia, severe insulin resistance requiring high doses of insulin to control these elevated blood glucose levels, increased ketosis and especially the hyperosmolar hyperglycemic state (nonketotic) (HHS) in older individuals suggest that COVID-19 is capable of having diabetogenic effects that exceed the well-accepted and recognized stress response associated with severe illnesses [5].

Therefore, it is crucial to better understand the longitudinal morphological changes of islet remodeling, which include (i) systemic and islet RAAS activation; (ii) islet redox stress; (iii) systemic and islet inflammation; (iv) islet amyloid; (v) islet fibrosis and (vi) β-cell dysfunction and/or failure due to loss (apoptosis) and capillary rarefaction. Interestingly, newer terminology has already been suggested to describe emerging conditions associated with this pandemic such as “COVID toes” (a clinical finding of erythematous to purple purpuric macules, papules, which resemble pernio-like, pseudo-chilblain acute acro-ischemia, supporting a role of these findings and an association with COVID-19) [6] and “COVID-19 pericyte hypothesis” (an immunohistologic finding demonstrating the presence of the ACE2 receptor on pericytes, which supports an important role of the pericyte in association with COVID-19 and damage to the barrier function of the endothelial cells) [7]. This novel terminology suggests that in due time we may come to know of the long-term complications of COVID-19 including T2DM and refer to this sequela as “COVID-19-related T2DM”.

In an effort to demonstrate how MetS and T2DM may have already damaged the islet prior to COVID-19 infections, multiple images generated by light microscopy and ultrastructure transmission electron microscopy (TEM) in preclinical MetS, T2DM rodent models and humans will be utilized to demonstrate islet injuries and the response to injury remodeling. Additionally, there seems to be a definite bidirectional relationship between COVID-19 and MetS/T2DM.

## 2. Systemic and Islet Renin–Angiotensin–Aldosterone System (RAAS) Activation in MetS, T2DM and COVID-19

**Figure 4:**
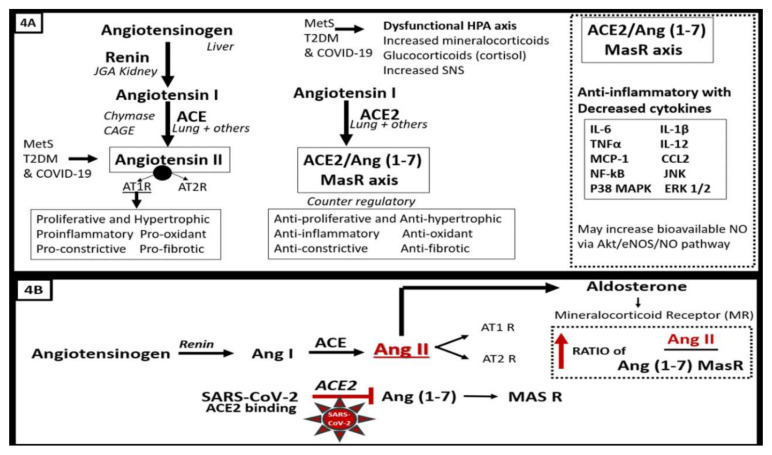
(**A**,**B**). The protective role of the ACE2–Ang (1–7)–MasR axis is attenuated and/or Lost in COVID-19 due to SARS-CoV-2 binding of ACE2 in pancreatic islets. Since SARS-CoV-2 in COVID-19 infections will bind to the ACE2 enzyme in order to gain entry into the β-cells once viremia has occurred, the anti-inflammatory effects will be less available to help prevent the proinflammatory effects of ACE/Ang II and inflammation and fibrosis will prevail in the peri-islet and intra-islet regions along with the deposition of islet amyloid polypeptide (IAAP) and collagen types I and III (fibrosis). The binding of SARS-CoV-2 to ACE2 in the islet β-cells may be partially responsible for the elevated blood glucose during the acute/immediate phase of COVID-19 and also the increased insulin resistance that is known to occur in individuals with COVID-19. Further, in the convalescent period and into the late-complication phase of COVID-19, there may be an increased risk for the development of T2DM and progression from orally treated T2DM to an insulin-dependent type of T2DM. Therefore, the loss of the ACE2–Ang (1–7)–MasR axis is of critical importance in COVID-19 [19]. Dashed boxes indicate the loss of the ACE2–Ang (1–7)–MasR axis and their protective pathways. ACE = angiotensin-converting enzyme; AGE = advanced glycation end products; Ang II = angiotensin II; CAGE = chymostatin-sensitive angiotensin II-generating enzyme; ERK CCL2 = C-C motif chemokine 2; ERK 1/2 = extracellular signal-regulated kinase 1/2; HPA = hypothalamic–pituitary–adrenal axis; IL-1β = interleukin-1beta; IL-6 = interleukin-6; IL-12 = interleukin-12; JNK = c-Jun N-terminal kinase; MasR = MAS-related G protein-coupled receptor; MCP-1 = monocyte chemoattractant protein 1; NF-KB = nuclear factor-kappa B; p38 MAPK = p38 mitogen-activated protein kinase; RAGE = receptor for AGE; RONS = reactive oxygen and nitrogen (nitrosative stress) species; TNFα = tumor necrosis factor alpha.

There is considerable evidence of an activated systemic or circulatory (cRAAS) as well as a localized tissue (tRAAS) in the pancreatic islet and also the epithelial ductal cells of the exocrine pancreas (Figure 1, Figure 2, Figure 3 and Figure 4A,B) [8,9,10,11,12,13,14,15,16,17,18]. The normal sequence of the classical cRAAS and tRAAS cascade begins with the activation of juxtaglomerular apparatus in the kidney due to several different stimuli such as beta-1 adrenergic stimulation, decreased renal perfusion pressure and decreased sodium chloride concentration, where it acts on angiotensinogen synthesized in the liver to produce angiotensin I. Angiotensin I is then acted upon by angiotensin-converting enzyme (ACE) found in multiple tissues including the lung and is converted to angiotensin II (Ang II), which has multiple effects on tissues that can become harmful or pathologic if it is chronically and excessively produced as occurs in MetS and T2DM (Figure 4A,B) [8,9,10,11,12,13,14,15,16,17]. Pathologic effects of Ang II such as activation of reduced nicotinamide adenine dinucleotide phosphate oxidase (NADPH oxidase) with increased superoxide and reactive oxygen and nitrogen (nitrosative stress) species (RONS) and proliferative, hypertrophic, proinflammatory, pro-constrictive and profibrotic effects within the local pancreatic islet tissues play an important role in β-cell dysfunction and loss (Figure 4A,B). 

Importantly, the RAAS cascade also has a built in counter-regulatory mechanism or a protective aspect in that angiotensin I may be converted to a protective side of the RAAS cascade via the ACE2 enzyme to Ang (1–7) → Mas-related G protein-coupled receptor (MasR) (commonly referred to as the ACE2/angiotensin (1–7) Mas receptor axis). This axis is known to be present on pulmonary pneumocytes as well as numerous other tissues including the pancreatic islet β-cell and peri- and intra-islet capillary mural endothelial cell(s) (EC) and possibly pericyte(s) (Pc), as recently found in brain and myocardial tissues (Figure 4A,B) [4,16,18,19].

**Figure 5:**
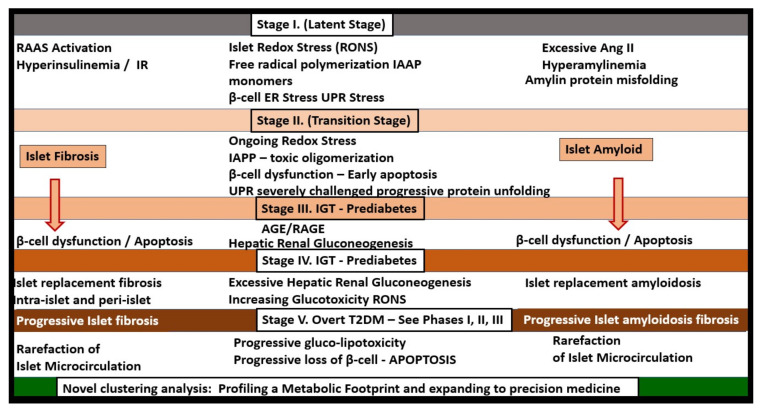
T2DM is a progressive disease consisting of five stages in the natural history of T2DM. T2DM implicates metabolic syndrome, redox stress, islet amyloid, islet fibrosis and RAAS. T2DM is the end stage of a process that involves a definite loss of pancreatic β-cell function and β-cell apoptosis that has multiple stages (I–V) during its development of this end-stage isletopathy. This figure represents a putative model of these five stages and how they are associated with islet amyloid, islet fibrosis and involves not only a systemic cRAAS but also a local tRAAS with excess islet Ang II production. Most often, insulin resistance is associated early on in this process and relates to a central role or state of hyperinsulinemia and hyperamylinemia as noted in metabolic syndrome in Figure 1. Loss of β-cell function and/or loss via apoptosis eventually develops and blood glucose levels continue to rise. Stages I–V demonstrate that T2DM is a progressive disease as in Figure 3, which involves islet amyloid and islet fibrosis remodeling. Thus, the late complication of T2DM development in previous non-diabetic patients may depend on the stage of development of the individual when they develop COVID-19 in relation to the type of T2DM during the development of late complications since SARS-CoV-2 may either involve the islet and its β-cells directly or indirectly. Further, an individual that is controlled on oral medication and lifestyle modifications may indeed develop an insulin-dependent type of T2DM due to the acceleration of the underlying stage at the time of infection due to loss of β-cells and possibly due to novel hybrid forms of diabetes such as latent autoimmune diabetes in adults following COVID-19. T2DM is a heterogeneous, multifactorial spectrum disease. Therefore, not all individuals who develop T2DM will strictly follow this 5-stage roadmap in a lock-step manner. Importantly, recent findings utilizing clustering analysis for the development of T2DM and its complications are being utilized and may relate to the personalized treatment of various clusters in addition to stages I–V. See Section 8, T2DM May Be Considered a Spectrum Disease. The novel clustering analysis that is currently being utilized will add a great deal of knowledge to stages I–V [27,28,29]. ANG II/Ang II = angiotensin II; AGE = advanced glycation end products; ER = endoplasmic reticulum; IAPP = islet amyloid polypeptide; IGT = impaired glucose tolerance; IR = insulin resistance; RAAS = renin–angiotensin–aldosterone system; RAGE = receptor for AGE; RONS = reactive oxygen and nitrogen (nitrosative stress) species; UPR = unfolded protein response.

**Figure 6:**
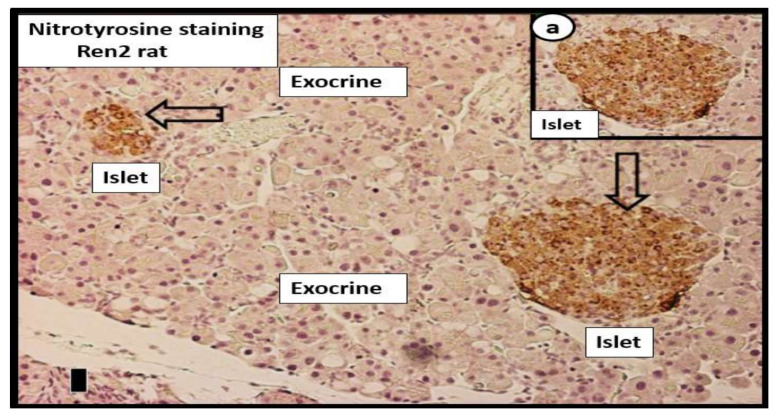
Immunohistochemistry of 3-nitrotyrosine staining in the Ren2 lean hypertensive preclinical rat model. This representative image demonstrates the strong diffuse antibody staining for 3-nitrotyrosine (a direct marker of nitrosative stress and an indirect marker of oxidative stress (RONS) in two islets (open arrows) in the 10-week-old transgenic Ren2 model (Ren2C) pancreas indicating intra-islet oxidative stress. Note that there is no staining in the adjacent exocrine portion of the pancreas. Insert **a**. demonstrates the diffuse intra-islet staining of 3-nitrotyrosine in the Ren2 model. Importantly, there was no staining for 3-nitrotyrosine in islets or exocrine portion of the age-matched Sprague–Dawley control model (not shown). Magnification: ×20 objective; scale bar = 5 µm. Courtesy JOP [22]. RONS = reactive oxygen and nitrogen (nitrosative stress) species.

In MetS and T2DM, the cRAAS and tRAAS of pancreatic islets are activated and play an important role in the progression of the natural history of T2DM (Figure 3, Figure 4A,B and Figure 5) [18,19]. Importantly, both cRAAS in ECs/Pcs and tRAAS in the pancreatic islet capillary ECs are activated with further activation by SARS-CoV-2, as it binds to the ACE2 receptors and diminishes its effect during COVID-19 infections. The binding of SARS-CoV-2 to ACE2 receptors will also contribute to further activation of the ECs/Pcs, which is detrimental to the integrity of the ECs at the microvascular regions [18,19,20]. From the alveolar capillary unit blood gas barrier in pulmonary tissues to pancreatic islets, the capillary ECs are activated and may contribute to and be associated with the attenuation or loss of the endothelial glycocalyx (ecGCx) [21]. The ecGCx is also important to the integrity of the capillary ECs and the end-organ tissue parenchyma it supplies as in the pancreatic islet and could contribute to the dysfunction and damage of β-cells in addition to the activation of cRAAS and the local pancreatic islet tRAAS associated with MetS and T2DM [21]. Ang II is the most potent activator of the NADPH oxidase in addition to aldosterone, which results in a marked increase in islet oxidative redox stress (Figure 4A,B). For example, in the transgenic Ren2 rat preclinical model of hypertension, tissue RAS overexpression with increased renin and Ang II (10 weeks of age) demonstrated that only the islets were stained excessively with 3-nitrotyrosine as compared to the Sprague–Dawley control age-matched controls (Figure 6) [18,19]. Further, the involvement of the exocrine pancreatic ductal cells via SARS-CoV-2 binding to the ACE2 receptor may interfere with the islet–exocrine interface within the pancreas [22,23]. Recently, the first reported case of acute pancreatitis in a COVID-19 individual was reported and in a recent case series of 52 COVID-19 patients, pancreatic injury (defined by any abnormalities in amylase/lipase) was present in up to 17% of patients [24,25,26]. Therefore, both the pancreatic endocrine and exocrine tissues may be affected by SARS-CoV-2 in COVID-19 infections.

Incidentally, the insulin resistance and hyperinsulinemia in stage I (Figure 5) also play a central role in MetS (Figure 1) as well as an early role in the development of T2DM. Indeed, hyperinsulinemia is capable of driving insulin resistance [30,31,32].

## 3. Islet Redox Stress in MetS, T2DM and COVID-19 

MetS and T2DM are complicated and progressive without drug treatment or lifestyle changes and are associated with multiple metabolic toxicities, resultant reactive oxygen and nitrogen (nitrosative stress) species (RONS), and end-organ islet remodeling. As these two abnormalities intersect with COVID-19, there may be even greater oxidative stress as these diseases merge and undergo significant crosstalk [5,26]. The excessive RONS and the resultant impairment in antioxidant counter-regulatory mechanisms create an imbalance due to antioxidant depletion within pancreatic islets and β-cells (Figure 6). This concept is not to be underestimated, especially once infected with SARS-CoV-2, with the known lack of β-cell antioxidative reserves [33,34,35,36].

**Figure 7:**
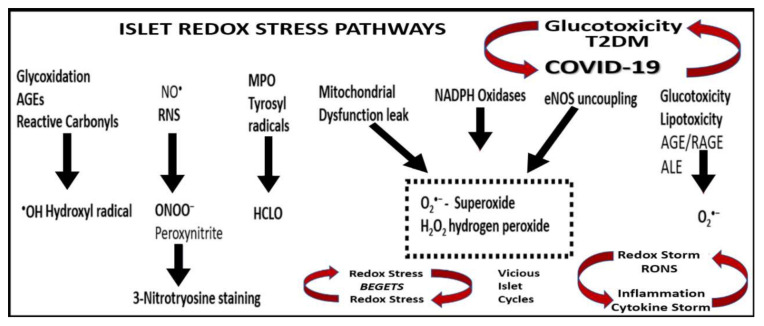
Islet reactive oxygen and nitrogen (nitrosative stress) species: possible pathways and enzymes responsible for the production of primary and secondary RONS in MetS, T2DM and COVID-19. The possible metabolic pathways and enzymes involved in the production of islet reactive oxygen and nitrogen (nitrosative stress) species (RONS) are illustrated. Both primary and secondary RONS are present in MetS, T2DM and COVID-19; however, it is the secondary RONS that act to produce RONS damage to tissues specifically in the pancreatic islets and consist of primary species (primary RONS), which react with one another or a transition metals (such as iron in Fenton reactions), yielding highly reactive secondary species, such as ONOO^−^ or ^•^OH, and may react to produce the elevated ferritin levels in Figure 1. With the abundance of redox stress and accumulation of RONS, one could consider the use of the term “redox storm”. Redox stress begets redox stress and reactive oxygen and nitrogen (nitrosative stress) species beget inflammation via NF-κB, creating at least two vicious cycles within islets in addition to the vicious cycle between T2DM/glucotoxicity and COVID-19. AGE = advanced glycation end products; ALE = advanced lipoxidation end products; eNOS = endothelial nitric oxide synthase; H_2_O_2_ = hydrogen peroxide; HCLO = hypochlorous acid; MPO = myeloperoxidase; NADPH = nicotinamide adenine dinucleotide phosphate reduced; NF-κB = nuclear factor-kappa B; NO^•^ = nitric oxide; RAGE = receptor for AGE; RONS = reactive oxygen and nitrogen (nitrosative stress) species.

The hyperglycemic glucotoxicity that is noted in COVID-19 patients and in T2DM is capable of producing even greater amounts of reactive oxygen and nitrogen (nitrosative stress) species via multiple effects, which include (*i*) glucose autoxidation; (*ii*) the polyol and hexose flux pathway; (*iii*) the Schiff reaction during glycation, resulting in advanced glycation end products (AGE) and its receptor (RAGE) interaction; (*iv*) mitochondria ROS due to excessive oxidative phosphorylation in the mitochondria with an excessive electron leak of unpaired electrons and oxidative phosphorylation during anaerobic glycolysis; (*v*) eNOS uncoupling; (*vi*) NADPH oxidase activation not only within the islet vasculature but also in the islet β-cell itself via a protein kinase C-dependent mechanism. This elevated tension of islet redox stress, coupled with low endogenous antioxidant activity within the islet allows ROS damage to proteins, nucleic acids, and lipids in the islet and the β-cell. Additionally, RONS may result in damage and dysfunction to messenger proteins, nuclear proteins, and plasma membranes, leading to impaired insulin gene expression, signaling, secretion and ultimately β-cell dysfunction and/or apoptosis [20,37]. One might even term this excessive redox stress as a “redox storm” that is present in addition to the more commonly new term in regard to COVID-19, a “cytokine storm” (an out-of-proportion excessive hyperinflammation phenotype or hypercytokinemia) (Figure 7) [21,38].

## 4. Systemic and Islet Inflammation

MetS and T2DM are thought to be associated with inflammatory dysregulation and are thought to be chronic inflammatory states [39,40,41,42,43]. Much of this inflammation is thought to be due to the accumulation, hypertrophy and rupture of adipocytes and inflammation of the visceral adipose tissue [37,38,39,40]. It is not only increasingly recognized that a low-grade systemic inflammation precedes and predicts the development of both T2DM and CVD but currently this concept is well established in the medical and research community [39,40,41,42,43,44]. Additionally, COVID-19 has been shown to have a dysregulated adaptive immune system response to SARS-CoV-2 and develop a novel COVID-19 cytokine storm [45]. The typical cytokines that are involved in the cytokine storm of COVID-19 may include the interleukins (IL-6, IL-1β, IL-12, and IL-15), tumor necrosis factor alpha (TNFα), chemokine (C-C motif ligand 2 (CCL2)/monocyte chemoattractant protein-1 chemokine (MCP-1)), c-Jun N-terminal kinases (JNK), which belong to the mitogen-activated protein kinase family, and are responsive to stress stimuli, such as cytokines, nuclear factor-kappa B (NF-κB), which mediates induction of pro-inflammatory cytokines, chemotactic factors and adhesion molecules, thereby promoting monocyte recruitment and downstream cytokines/chemokines, extracellular signal-regulated kinases/p38 mitogen-activated kinases (ERK ½—p38 MAPK), signaling proteins that are sensitive to ACE2–Ang(1–7)–MasR axis anti-inflammatory actions (Figure 4) [19]. Both the activation of a cRAAS and tRAAS associated with excessive ANG II generation and a redox storm promote inflammation (Figure 1, Figure 3, Figure 4, Figure 6 and Figure 7). Additionally, the redox storm begets an inflammatory storm via NF-κB and the inflammatory storm begets a redox storm and reactive oxygen and nitrogen (nitrosative stress) species (RONS) beget RONS, creating vicious cycles within the islets with β-cell dysfunction and loss via apoptosis (Figure 7) [46]. While inflammation is an essential part of an effective immune response, which is normal in the wound healing response to infections that normally resolve and return to homeostasis, SARS-CoV-2 induces excessive and prolonged cytokine/chemokine responses by causing an impaired and dysregulated immune system, resulting in a cytokine storm [47]. Chronic ongoing stimulation due to the cytokine storm in SARS-CoV-2 may result in chronic inflammation, which is thought to result in the elevated ferritin, as in Figure 1, and result in many damaging remodeling changes such as chronic fibrosis that may affect pancreatic islets and β-cell function and loss via apoptosis.

## 5. Islet Fibrosis

**Figure 8:**
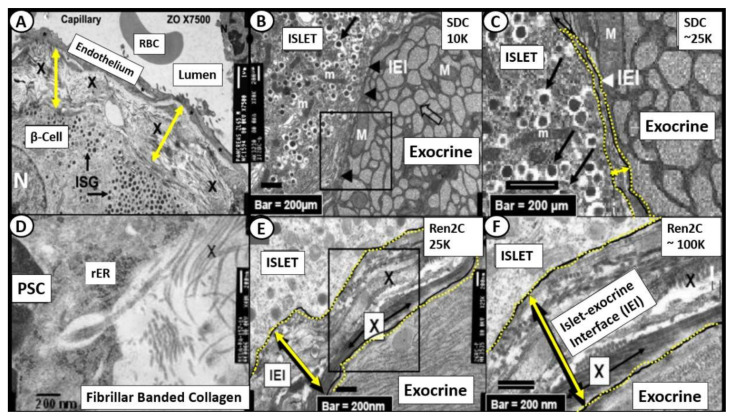
Intra-islet pericapillary and peri-islet (islet-exocrine interface) fibrosis. (Panel (**A**)) depicts the widening and early peri-capillary fibrosis in the Zucker obese male model of insulin resistance, obesity and impaired glucose tolerance at 12 weeks of age. (Panels (**B**,**C**)) demonstrate the close association of the islet-exocrine interface, with only slight widening at even high magnification between the exocrine and endocrine pancreas in the male control Sprague–Dawley control (SDC) model at 10 weeks of age. (Panels (**E**,**F**)) demonstrate a marked widening at higher magnification as compared to the SDC model in B and C in addition to the accumulation of collagen (X). Importantly, (Panel (**D**)) depicts the extrusion of collagen from a pancreatic stellate cell (PSC) in the Ren2 model at 10 weeks of age; scale bar = 200 nm. IEI = islet-exocrine interface; ISG = islet secretory granule; M and m = mitochondria; PSC = pancreatic stellate cell; RBC = red blood cell; rER = rough endoplasmic reticulum; X = collagen within the widened IEI.

**Figure 9:**
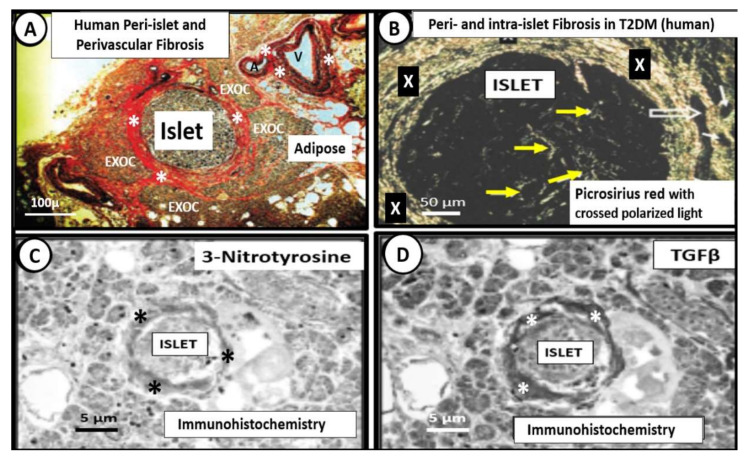
Human intra-islet, peri-islet and perivascular fibrosis. (Panels (**A**,**B**)) depict peri- and intra-islet fibrosis and perivascular fibrosis in a female human (age 58) with T2DM of 6 years duration who was insulin dependent for one year at time of death from an acute myocardial infarction. (Panel (**A**)) depicts marked peri-islet, and perivascular fibrosis in the pancreas and thus is capable of interfering with communication between the islet endocrine and exocrine pancreas with Verhoeff Van Gieson (VVG) staining for fibrosis (crimson-red) (asterisks) and elastin (black). Note perivascular fibrosis surrounding an arteriole (A) and venule (V) (asterisks). (Panel (**B**)) demonstrates marked peri-islet fibrosis (X) and intra-islet fibrosis (yellow arrows). Note that the open arrows depict the islet–acinar–portal vascular pathway (venule) utilizing picrosirius red staining with crossed polarized light. (Panels (**C**,**D**)) represent the immunohistochemistry staining for 3-nitrotyrosine (reflecting oxidative nitrosative stress) and TGFβ (profibrotic cytokine-growth factor), respectively. Images A–D modified via courtesy of [48,52]. EXOC = exocrine pancreas.

Islet fibrosis is related to systemic (cRAAS) and islet RAAS (tRAAS) (Section 2), islet redox stress and RONS (Section 3) and systemic and islet inflammation (Section 4). Islet fibrosis is a common finding early on in MetS and T2DM, which is found not only in preclinical animal models (Figure 8) but also in adult humans (Figure 9) [20,22,48,49,50,51,52,53,54]. The pancreatic stellate cell is important [48,50] and may reflect the activation of the peri-islet pericytes in the Ren2 model of lean hypertension with excess renin and Ang II (Figure 8). Currently, we do not know whether those infected with SARS-CoV-2 will develop islet fibrosis; however, the pre-existing conditions certainly suggest that this will be a common mechanism should the response to wound injury healing mechanisms not subside. Interestingly, we have previously observed that islet fibrosis and islet amyloidosis may be concurrent mechanisms especially in the peri- and intra-islet locations in various animal models and could represent a mechanism that could be accelerated in those individuals infected with COVID-19 due to chronic and ongoing inflammation and fibrosis in the post-recovery period (Figure 8 and Figure 9).

## 6. Islet Amyloid/Amylin/Islet Amyloid Polypeptide (IAPP) 

Knowledge of a space-occupying lesion (originally described as hyalinosis) within the pancreatic islets of T2DM patients (currently known as islet amyloid/islet amyloid polypeptide (IAPP)) has been known for over a century [55]. However, it is often not discussed as an integral part of the natural history of T2DM in humans (Figure 1, Figure 2 and Figure 5) [55,56]. Amylin (IAPP) is a 37 amino acid β-cell-derived hormone that is co-synthesized and co-packaged in the endoplasmic reticulum within the insulin secretory granule (ISG) of the Golgi apparatus, which is then co-secreted with the ISG along with insulin from the β-cells of the pancreatic islets into the systemic circulation. In situations of insulin resistance, as occurs in MetS with associated compensatory hyperinsulinemia, the β-cells will also synthesize and secrete greater amounts of amylin, which will result in hyperamylinemia (Figure 1), with subsequent islet amyloid deposition [56,57,58,59,60,61,62,63].

Amyloid deposition within the pancreatic islets of MetS and T2DM (islet amyloidosis) occurs primarily in humans, feline species and non-human primates but does not appear in rodents due to the lack of amyloidogenic rodent amylin because of a proline substitution at positions 25, 28, and 29 of the 37 amino acid amylin. Interestingly, T2DM does not occur in rodent models without transgenic manipulation. These transgenic models include the human islet amyloid polypeptide gene transfection in the HIP rat model (as presented in this review) or in mice. Additionally, spontaneous genetic abnormalities occur in the obese insulin-resistant ob/ob mouse and rat, BTBR ob/ob and db/db models of obesity, insulin resistance and T2DM.

**Figure 10:**
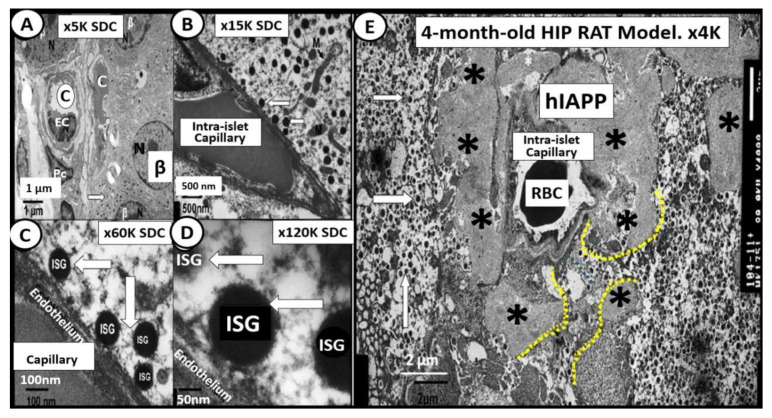
Normal insulin secretory granule docking in control and impaired docking to the capillary endothelium in the HIP rat models. Panels (**A**–**D**) illustrate the close association of β-cells to the islet capillaries, which allow for proper docking of the insulin secretory granule (ISG) (white open arrows) for absorption of insulin in the 4-month-old control Sprague–Dawley control (SDC). Magnifications and scale bars are in each image. Panel (**E**) depicts the diffuse islet amyloid deposition (asterisks) in the 4-month-old HIP rat model. This image allows one to note the impairment of ISG docking with the islet capillary. Note only the small spaces (outlined by yellow dashed lines) that are able to allow for minimal docking of ISGs. Note how the ISGs come to the peri-capillary islet amyloid and appear to be abruptly quarantined without access to the islet capillaries. These findings would fall into stages II and III of Figure 5 in Section 1. Magnification ×4000; scale bar = 2 µm. Asterisks = islet amyloid; C= capillary; hIAPP = human islet amyloid polypeptide; ISG = insulin secretory granule; K = 1000; M = mitochondria; RBC = red blood cell.

**Figure 11:**
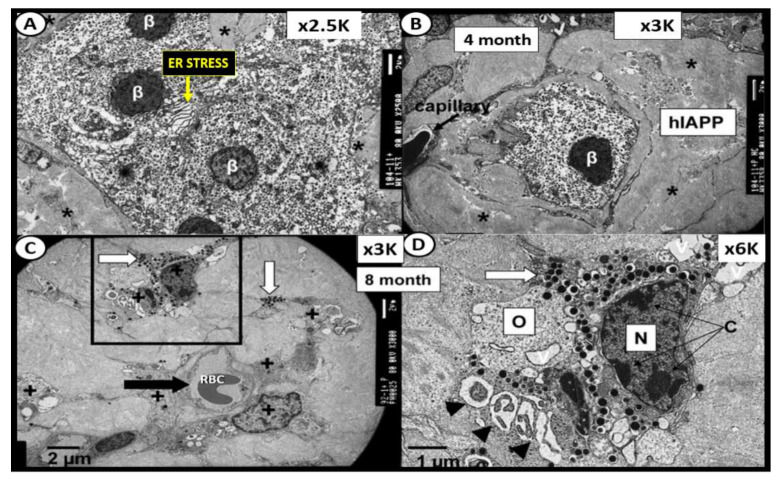
The 4- and 8-month-old HIP rat. (Panels (**A**,**B**)) from the 4-month-old HIP rat models. Panel (**A**) illustrates endoplasmic reticulum (ER) stress with prominent widening of the ER in β-cells (yellow arrow) and also note the islet amyloid (asterisk) in left lower region. Panel (**B**) depicts a single β-cell embedded and totally isolated by islet amyloid/human islet amyloid polypeptide (hIAPP) amyloidosis in 4-month-old HIP rat models. Panels C and D are from the 8-month-old HIP rat model. (Panel (**C**)) displays the marked decrease in β-cells, which are atrophic and represent changes of apoptosis. Note the islet capillary with its red blood cell (RBC) and its close association with an atrophic apoptotic β-cell with apoptotic bodies and loss of insulin secretory granules (black arrow). (Panel (**D**)) depicts an apoptotic β-cell from boxed in area in panel (**C**) and note the apoptotic bodies (arrowheads), loss of cytoplasmic organelles (O) and cytoplasmic vacuoles (apoptotic bodies) along with its nucleus (N) depicting chromatin condensation (C). β = β-cell; K = 1000; N = nucleus; O = cytoplasmic organelle; RBC = red blood cell.

**Figure 12:**
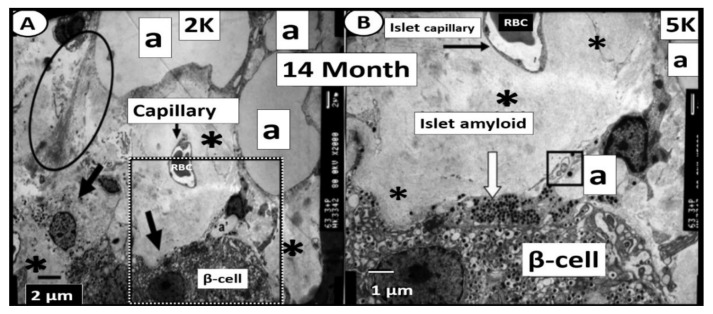
β-cell loss and near-complete islet amyloid replacement in 14-month-old HIP rat islets. There were very few β-cells remaining in 14 month-old models and most islets demonstrated near-complete replacement with islet amyloid and fatty infiltration of islets was prominent with large lipid droplets (a) in 14-month-old models in (Panels (**A**,**B**)). Oval in panel (**A**) encloses an early change in intra-islet fibrosis. Remaining β-cells were atrophic and capillaries and pericytes were decreased. The remaining pericytes demonstrated multiple lipid droplets in their cytoplasm similar to lipid droplets within the islets (not shown). Panel (**B**) is a higher mag of the dashed boxed in region in panel (**A**) [65]. Magnification ×2000 and ×5000 in Panels (**A**,**B**), respectively; scale bar = 2 µm in panel (**A**) and 1 µm panel (**B**). a = adipose lipid droplets; open and closed arrows = β-cell; K = 1000.

**Figure 13:**
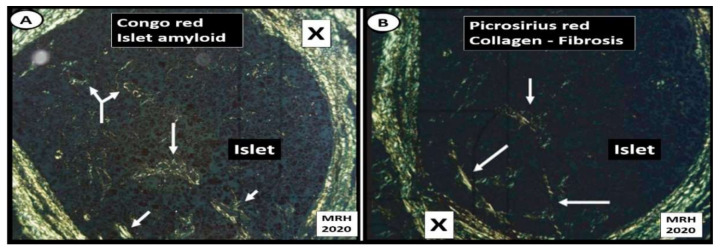
Islet amyloid and islet fibrosis in human islets co-occur. (Panel (**A**)) depicts Congo red staining (stains amyloid) viewed with crossed polarized light and birefringent appearance at the peri-islet (X) and intra-islet regions (arrows). (Panel (**B**)) illustrates islet fibrosis (collagen I and III) staining with picrosirius red and viewed with crossed polarized light of pancreatic islet at the peri-islet (X) and intra-islet regions (arrows). These images demonstrate that islet amyloid and islet fibrosis co-occur within human pancreatic islets when stained specifically for amyloid (Congo red) and fibrosis (picrosirius red) in a female patient who died of an acute myocardial infarction [54]. Specific staining procedures by pathologists often reveal what standard hematoxylin and eosin (H&E) staining do not in regard to conditions such as islet amyloid and fibrosis. Magnification ×100 objective with oil immersion in Panels (**A**,**B**).

The human islet amyloid polypeptide (HIP) rat model was created by the transfection of Sprague–Dawley rat with the human islet amyloid polypeptide (hIAPP)-amylin gene in 2004 and initially studied by Butler AE et al. [64]. This HIP rat model developed T2DM spontaneously on a normal rat chow diet and our group followed islet remodeling with ultrastructure studies at 2, 4, 8 and 14 months of age (Figure 10, Figure 11 and Figure 12) [65,66]. Additionally, the author was able to demonstrate the co-occurrence of islet amyloid and islet fibrosis in the pancreatic islets of a 58-year-old female patient with known T2DM who died of an acute myocardial infarction (Figure 9A,B and Figure 13) [54].

When SARS-CoV-2 binds to the β-cell ACE2 within pancreatic islets, they may also create novel lysosomal organelle membrane channels, which allow increased calcium transients into lysosomes, resulting perturbations of lysosomal contents within the β-cell as a result of protein oligomerization similar to how the IAPP amylin oligomers create channels within the plasma membrane of β-cells (Figure 2) [67]. The novel coronavirus, SARS-CoV-2, may encode ion-channel proteins called viroporins (protein E, open reading frame 3a (ORF3a) and ORF8a). These viroporins may induce lysosomal disruption and ion redistribution in the intra-cellular environment, which may activate the innate immune signaling receptor NOD-, LRR-, pyrin domain-containing 3 (NLRP3) inflammasome. This could not only lead to islet amyloid formation due to not destroying the misfolded proteins of amylin-IAPP within intact lysosomes but also result in increased islet amyloid polymerization to mature fibrils and deposition in the extracellular space within islets. The NLRP3 inflammasome activation mechanism could also activate the production of inflammatory cytokines such as interleukin 1β (IL-1β), IL-6 and tumor necrosis factor (TNFα), which could contribute to islet inflammation, as previously discussed (Section 4) [67]. Further, inadequate lysosomal degradation of misfolded IAPP may increase the vulnerability of β-cells to IAPP oligomer toxicity in addition to islet inflammation [68]. While one can view the oligomeric toxicity of islet amyloid within the cell, which involves the proper function of the unfolded protein response being impaired in T2DM, there is also the recent reasoning that the toxic oligomers of islet amyloid may occur in the ECM of the pancreatic islets and that it is here that these oligomers are even referred to as the pancreatic islet β-cell assassin [69].

It is important to point out that there currently exists some controversary as to whether or not ACE2 is present on or in the pancreatic islet β-cells. Two papers have recently been published demonstrating the lack of ACE2 enzyme receptors on β-cells [70,71], while there are two other papers suggesting that the ACE2 enzyme is present on β-cells [72,73]. Fignani D. et al. were able to not only demonstrate ACE2 staining in islet β-cells but were also able to demonstrate that inflammatory cytokines (Il-1β, IFNγ and TNFα) were capable of upregulating ACE2 in islet β-cells and thus support the importance of cytokine storm (hyperinflammation) and/or cytokine release syndrome in COVID-19 [73]. Hopefully, there will be some consensus regarding this important issue in regard to the development of both T1DM and T2DM in the coming months. In this regard, it is important to note that there is currently a general consensus that the microvascular EC/Pcs (both endocrine islet and exocrine) and exocrine ductal epithelial within the pancreas do stain positive for ACE2 [70,71,72,73]. 

## 7. Islet β-Cell Dysfunction and Failure Due to Loss (Apoptosis) and Capillary Rarefaction

**Figure 14:**
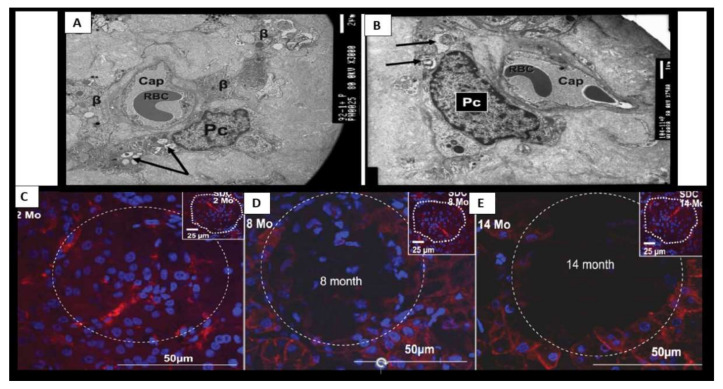
Pericyte apoptosis and loss within islets of 14-month-old HIP rat models. (Panels (**A**,**B**)) depict sparse islet capillaries embedded within a sea of islet amyloid. Note pericyte(s) (Pc) apoptosis with apoptotic bodies (arrows). Endothelial apoptosis was not observed in these intra-islet regions in the HIP rat models in contrast to Pcs. (Panels (**C**,**D**)) are confocal immunohistochemistry stains of the islets with antibodies to the platelet-derived growth factor receptor beta (PDGFR-β) (red) and DAPI (blue) staining of islet cells. Note the progressive loss of intra-islet staining of pericytes (PDGFR-β red stain) and also note the progressive loss of cellularity (DAPI blue staining) within the islets from two months to 14 months in the HIP rat models. This suggests that, in addition to the loss of intra-islet cellularity, there is a loss of capillary pericytes. Thus, pericyte loss/apoptosis appears to play a significant role in the development of T2DM, at least in the HIP rat model of T2DM, similar to that found in the retinas of T2DM diabetic preclinical models and humans. Note the inserts in the upper right of the Sprague–Dawley Control (SDC) models in (Panels (**C**–**E**)). The white dashed circle outlines the pancreatic islet margins in each of the images (**C**–**E**). Various magnifications are used, with scale bars readily identified in each image. Scale bar = 2 µm in panel (**A**) and 1 µm panel (**B**).

β-cell dysfunction contributes to impaired secretion of insulin in MetS and T2DM [74,75,76]. More importantly, there is accumulating evidence that B-cell apoptosis is a serious threat to the development of T2DM that may be characterized by β-cell atrophy, chromatin condensation, inter-nucleosomal DNA fragmentation, and disassembly into membrane-encircled vesicles (apoptotic bodies) (Figure 11C,D) [77]. In addition to β-cell atrophy and apoptosis with the co-occurrence of islet amyloid and islet fibrosis, there was also noted to be islet capillary rarefaction in the 14-month-old HIP rat model (Figure 14). The islet capillaries are composed of both pericytes and endothelial cells, in which, each are dependent on the other for proper microvascular functioning, as it takes both of these cells for proper vascular supply and functioning within the pancreatic islets. This is quite similar to the important role of pericytes in diabetic retinopathy such that if the pericyte is dysfunctional or damaged, the endothelium also becomes dysfunctional and damaged, which results in pancreatic intra-islet capillary dysfunction and or loss as in capillary rarefaction [20,52,66]. Importantly, we need to stay connected to the evolving role of pericytes in the intra-islet capillaries since ACE2 enzyme (important for binding SARS-CoV-2) has been identified in both the brain and heart pericytes and additionally we already understand that pericytes are ubiquitous throughout the microcirculation including the pancreatic islets and play an important and supportive role in islet blood supply [7].

## 8. T2DM May Be Considered a Spectrum Disease

**Figure 15:**
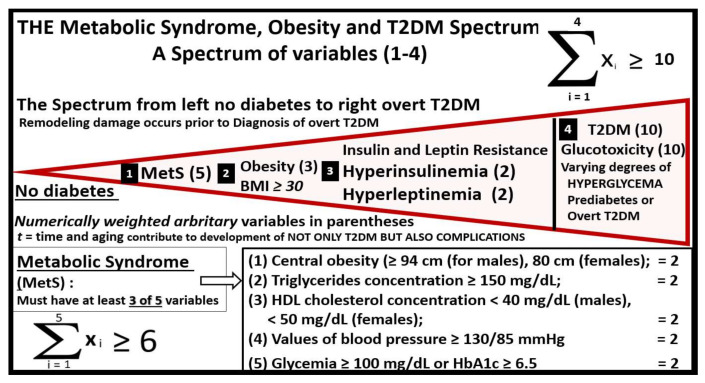
T2DM may be considered a spectrum disease. The development of overt or clinical T2DM could be considered a spectrum disease, in that, end-organ tissue remodeling in response to multiple metabolic toxicities begin long before the clinical diagnosis of T2DM is made; see Figure 1, Figure 3 and Figure 5. Note that there are multiple variables (black boxed-in numbers 1–3) that contribute to the development of T2DM and its associated multiple end-organ complications (including the pancreatic islet—isletopathy) even prior to the clinical diagnosis of overt T2DM (black boxed-in number 4). Importantly, there are multiple variables within metabolic syndrome (MetS) (black boxed-in number 1), which consist of having three of the following five ATP guideline variables consisting of: central obesity (≥94 cm (for males), 80 cm (females)); triglycerides concentration ≥ 150 mg/dL; HDL cholesterol concentration < 40 mg/dL (males), <50 mg/dL (females); values of blood pressure ≥130/85 mmHg and fasting glycemia ≥ 100 mg/dL or HbA1c ≥ 6.5 that contribute to its summation equation [8,9]. Lipotoxicity was not assigned a boxed-in number because HDL cholesterol and triglycerides variables are included in MetS (black boxed-in number 1) as one of the five criteria for diagnosing MetS. Hypertension may be one of the most common health problems associated with obesity and is included in MetS (black boxed-in number1) variables and is not within the scope of this paper to discuss in greater depth. The importance of insulin and leptin resistance (black boxed-in number 3) is central and may be critical to islet and even brain remodeling and therefore is not to be underestimated. Note the novel simple mathematical summation equations that are introduced with black boxed-in variables 1–4 and that the author has assigned various weighted numbers (based on their relative importance) in parentheses for summation equation totals, which points to the importance of each variable and their summation. The author is aware that that the weighted values of each of the variables utilized in these summation equations are debatable. ATP III = adult treatment panel III (ATP III) report of the National Cholesterol Education Program; HbA1c = hemoglobin A1C; HDL = high-density lipoprotein; MetS = metabolic syndrome; T2DM = type 2 diabetes mellitus.

T2DM is not only considered a heterogeneous, multifactorial, environmental (overnutrition and underexercise) polygenetic (both prenatal and adult) disease (Figure 1) with β-cell dysfunction and or loss via apoptosis [78] but can also be considered a spectrum disease, associated with a spectrum of variables (Figure 15).

Additionally, the above summation equations with multiple variables may be calculated from the patient’s past medical history, current medications regarding treatment for T2DM, vital signs including weight and height and current laboratory values upon admission utilizing the variables set forth in Figure 15 and contribute to our database of knowledge. These summation equations could possibly be incorporated as an algorithm and utilized in our deep learning technology to calculate and to save for future reference and further data input to aid in the understanding the convergence of these two diseases and how they interact. Of course, (t) for time or lag-time is important to understand in regard to the natural history of the progressive development of T2DM; however, it may turn out to be somewhat difficult to calculate this (t) for time in these summation equations (Figure 3 and Figure 15).

## 9. Glucotoxicity

**Figure 16:**
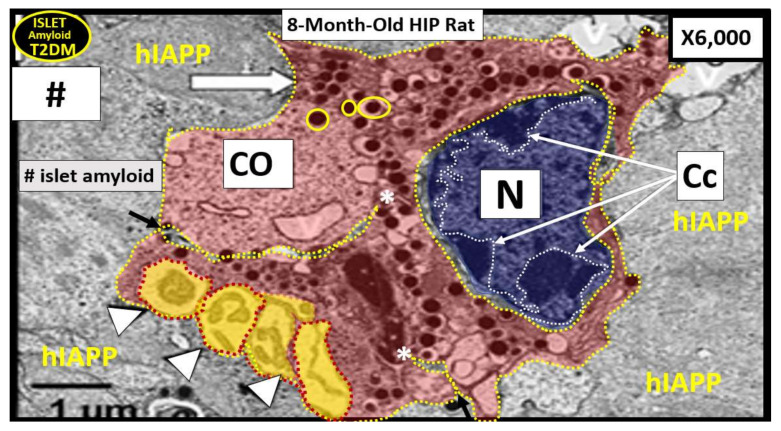
Pancreatic islet β-cell apoptosis in the HIP rat model of T2DM. This representative pseudo-colorized pancreatic islet β-cell in the preclinical 8-month-old HIP rat diabetic model (Figure 11D) depicts apoptosis characterized by β-cell atrophy, nuclear chromatin condensation (Cc with white arrows), loss of cytoplasmic organelles (CO), cytoplasmic vacuole formation and apoptotic bodies (arrowheads). Note how islet amyloid may insert into β-cells (arrows and asterisks). β-cell apoptosis in MetS, T2DM and COVID-19 may be due to a combination of a virus virion storm (SARS-CoV-2), redox storm and cytokine storm as they converge and interact with the multiple metabolic toxicities of MetS and prediabetes or overt T2DM, which may result in injury and a response to the injury wound healing mechanism within the islet and its microcirculation and encourage the acceleration of T2DM due to early dysfunction and later loss of β-cells via apoptosis. Nucleus (blue); cytoplasm (red); apoptotic bodies (yellow); extracellular matrix—hashtag islet amyloid (concrete grey color). CO = cytoplasmic organelles; hashtag = islet amyloid; hIAAP = human islet amyloid polypeptide; ISG = electron dense insulin secretory granules (open arrow and encircled by yellow lines of both immature haloed and more mature unhaloed ISG).

Glucotoxicity also becomes an important variable to consider in regard to pancreatic β-cell death via apoptosis (variable numbers 1, 4 in Figure 15) [79,80]. Wang S et al. has recently reported that a fasting blood glucose value that is ≥7.0 mmol/L (126 mg/dL) at admission in COVID-19 individuals was an independent predictor for 28 day mortality in patients with COVID-19 even without a previous diagnosis of diabetes [76]. Ultimately, the metabolic toxicities of MetS and T2DM (glucotoxicity, lipotoxicity, reactive oxygen and nitrogen (nitrosative stress) species (RONS), islet amyloid, inflammation and endoplasmic reticulum stress) may intersect with SARS-CoV-2 virions binding to the ACE2 receptor and may result in pancreatic islet β-cell failure due to apoptosis, as illustrated in the pseudo-colorized 8-month-old HIP rat model of T2DM and islet amyloid (Figure 16) [65,66,81,82]. Specifically, acute and chronic hyperglycemia results in compensatory pancreatic β-cell endoplasmic reticulum (ER) stress and is capable of resulting in β-cell dysfunction and apoptosis via the unfolded protein response (UPR) stress activation of proapoptotic pathways [83].

## 10. Conclusions

As the COVID-19 pandemic tears through coastal borders of countries, states, provinces, major cities and even small towns all over the world, it sadly results in mortality to some, but so far there has been a much greater number of survivors who may be at risk for long-term complications. While it is too early to know what specific long-term complications this pandemic may have or what specific organs COVID-19 damages in the long run, we must be prepared to better understand these possible long-term complications. However, the author’s interests concern the long-term complication of developing T2DM or worsening T2DM.

Sowers JR et al. were the first to sound an alarm regarding the intersection of diabetes and COVID-19 [84] and Rubino F et al. set forth the hypothesis that there may be a potential diabetogenic effect of COVID-19, beyond the well-recognized stress response of acute illness [5]. They further asked how frequently this phenomenon of new-onset diabetes may occur and importantly, asked whether COVID-19 might change the underlying pathophysiology and the natural history of diabetes [5]. This international group of leading diabetes researchers participating in the CoviDIAB project have established a global registry of patients with COVID-19–related diabetes (covidiab.e-dendrite.com), which will aid tremendously in how these two disparate diseases merge during this COVID-19 pandemic and the possible long-term complications.

This entire review set out to better understand the natural history of T2DM (Figure 1, Figure 3 and Figure 5) and discuss its pathophysiology and how COVID-19 might accelerate the natural history of T2DM due to SARS-CoV-2 binding to the ACE2 receptor on the islet endothelial/pericyte microvasculature and the pancreatic islet β-cells with an ensuing viral virion storm, redox storm and cytokine storm.

When one examines the co-morbidities associated with more severe complications including assisted ventilation, sepsis, cytokine storm, increased thrombotic risk and mortality, there seems to be a solid core of at least five co-morbidities including (i) hypertension, (ii) diabetes, (iii) cardiovascular disease (including coronary artery disease and cerebrovascular disease), (iv) older age [85] and (v) obesity [1]. Furthermore, when one examines MetS (Figure 1), at least four of these five variables are present except for time or older age and we know that aging also has an important association with MetS and T2DM (an age-related disease) [21]. This is the reason the author has chosen to utilize the association of MetS with T2DM throughout the review along with the known increased relative risk of 3.5–5.2 for incident T2DM with MetS [86].

Through the previous Sections, we can now better understand the natural history of T2DM and how COVID-19 may intersect to accelerate the development of post-COVID-19-incident T2DM or worsen pre-COVID-19 T2DM resulting in patients becoming insulin dependent as a long-term complication. This review has focused primarily on T2DM because it affects such a large number of people globally and is responsible for approximately 90–95% of all diabetes cases. Nevertheless, it is important to also include type 1 diabetes mellitus (T1DM), as it may have a lag-time of up to one year, since pancreatic islet β-cells are being damaged and lost due to the autoimmune destruction of β-cells, which may also be accelerated by COVID-19 due to the viral virion storm, the redox storm and especially the cytokine storm. Examining pancreatic islets and the development of autoreactivity diabetes (autoimmune T1DM) in regard to the apoptosis of islet β-cells can aid in a better understanding of the mechanism of how SARS-CoV-2 may bind to the ACE2 receptor, which results in β-cell apoptosis and a better understanding of T1DM and its interaction with COVID-19 [87]. Additionally, it has been demonstrated that insulin administration is protective of the ultrastructural abnormal remodeling changes to the pancreatic islet microcirculation in streptozotocin-induced T1DM mouse models [88].

Limitations to this review include the prematurity of not knowing what the long-term complications will be at this time and one can only speculate; however, if we understand the pre-COVID-19 T2DM natural history of progression, then we can better understand how T2DM may interact with the convergence of COVID-19 and SARS-CoV-2. Thus, it may be possible that we can be more involved and better know how to prepare and treat this possible long-term complication of COVID-19.

Central to the acceleration of the natural history of both T1DM and specifically T2DM is the binding of SARS-CoV-2 to the ACE2 receptor on both β-cells and vascular mural ECs and pericytes as a direct injury and response to injury wound healing mechanisms within pancreatic islets. The direct and subsequent indirect effects of SARS-CoV-2 may be the injury and response to the injury by the innate wound healing mechanisms that appear to be tightly woven with the associated virus virion storm, the redox storm and the cytokine storm. The multiple storms associated with COVID-19 may be singularly or synergistically involved in β-cell dysfunction and loss via apoptosis (Figure 16) in addition to islet microcirculation abnormalities including islet capillary rarefaction, islet hypoxia and abnormal islet remodeling) (Figure 14).

In regard to future directions, it will be interesting to follow the story of both the ACE2 enzyme and the transmembrane serine protease 2 (TMPRSS2) gene polymorphisms to observe whether there are any genotype alterations that may be associated with the development of the accelerated natural history in MetS, T2DM and COVID-19 specifically in different global subpopulations [89].

## References

[1] Cariou B., Hadjadj S., Wargny M., Pichelin M., Al-Salameh A., Allix I., Amadou C., Arnault G., Baudoux F., Bauduceau B. (2020). Phenotypic characteristics and prognosis of inpatients with COVID-19 and diabetes: The CORONADO study. Diabetologia.

[2] Bansal R., Gubbi S., Muniyappa R. (2020). Metabolic Syndrome and COVID 19: Endocrine-Immune-Vascular Interactions Shapes Clinical Course. Endocrinology.

[3] Kruglikov I.L., Scherer P.E. (2020). The Role of Adipocytes and Adipocyte-Like Cells in the Severity of COVID-19 Infections. Obesity.

[4] Zhang Y., Somers K.R., Becari C., Polonis K., Pfeifer M.A., Allen A.M., Kellogg T.A., Covassin N., Singh P. (2018). Comparative Expression of Renin-Angiotensin Pathway Proteins in Visceral Versus Subcutaneous Fat. Front. Physiol..

[5] Rubino F., Amiel S.A., Zimmet P., Alberti G., Bornstein S., Eckel R.H., Mingrone G., Boehm B., Cooper M.E., Chai Z. (2020). New-Onset Diabetes in Covid-19. N. Engl. J. Med..

[6] Hernandez C., Bruckner A.L. (2020). Focus on “COVID Toes”. JAMA Dermatol..

[7] He L., Mae M.A., Sun Y., Muhl L., Nahar K., Liébanas E.V., Fagerlund M.J., Oldner A., Liu J., Genové G. (2020). Pericyte-specific vascular expression of SARS-CoV-2 receptor ACE2—Implications for microvascular inflammation and hypercoagulopathy in COVID-19 patients. bioRxiv.

[8] Carlsson P.O. (2001). The renin-angiotensin system in the endocrine pancreas. JOP. J. Pancreas.

[9] Lau T., Carlsson P.O., Leung P.S. (2004). Evidence for a local angiotensin system and dose-dependent inhibition of glucose-stimulated insulin release by angiotensin II in isolated pancreatic islets. Diabetologia.

[10] Leung P.S. (2003). Pancreatic renin-angiotensin system: A novel target for the potential treatment of pancreatic diseases?. JOP J. Pancreas.

[11] Leung P.S., Carlsson P.O. (2001). Tissue renin-angiotensin system: Its expression, localization, regulation and potential role in the pancreas. J. Mol. Endocrinol..

[12] Leung P.S. (2007). The physiology of a local renin–angiotensin system in the pancreas. J. Physiol..

[13] Leung P.S., Chappell M.C. (2003). A local pancreatic renin-angiotensin system: Endocrine and exocrine roles. Int. J. Biochem. Cell Biol..

[14] Tahmasebi M., Inwang E.R., Vinson G.P., Puddefoot J.R. (1999). The tissue renin-angiotensin system in human pancreas. J. Endocrinol..

[15] Tikellis C., Wookey P.J., Candido R., Andrikopoulos S., Thomas M.C., Cooper M.E. (2004). Improved islet morphology after blockade of the renin-angiotensin system in the ZDF rat. Diabetes.

[16] Goossens G.H. (2012). The Renin-Angiotensin System in the Pathophysiology of Type 2 Diabetes. Obes. Facts.

[17] Luther J.M. (2014). Effects of aldosterone on insulin sensitivity and secretion. Steroids.

[18] Hayden M.R., Sowers K.M., Pulakat L., Joginpally T., Krueger B., Whaley-Connell A., Sowers J.R. (2011). Possible Mechanisms of Local Tissue Renin-Angiotensin System Activation in the Cardiorenal Metabolic Syndrome and Type 2 Diabetes Mellitus. Cardiorenal Med..

[19] Prestes T.R.R., Rocha N.P., Miranda A.S., Teixeira A.L., Simoes-E-Silva A.C. (2017). The Anti-Inflammatory Potential of ACE2/Angiotensin-(1-7)/Mas Receptor Axis: Evidence from Basic and Clinical Research. Curr. Drug Targets.

[20] Hayden M.R., Sowers J.R. (2007). Isletopathy in Type 2 Diabetes Mellitus: Implications of Islet RAS, Islet Fibrosis, Islet Amyloid, Remodeling, and Oxidative Stress. Antioxid. Redox Signal..

[21] Hayden M.R. (2020). Endothelial activation and dysfunction in metabolic syndrome, type 2 diabetes and coronavirus disease 2019. J. Int. Med. Res..

[22] Hayden M.R., Karuparthi P.R., Habibi J., Wasekar C., Lastra G., Manrique C., Stas S., Sowers J.R. (2007). Ultrastructural islet study of early fibrosis in the Ren2 rat model of hypertension. Emerging role of the islet pancreatic pericyte-stellate cell. JOP J. Pancreas.

[23] Hayden M.R., Sowers J.R. (2008). Pancreatic Renin-Angiotensin-Aldosterone System in the Cardiometabolic Syndrome and Type 2 Diabetes Mellitus. J. Cardiometabolic Syndr..

[24] Aloysius M.M., Thatti A., Gupta A., Sharma N., Bansal P., Goyal H. (2020). COVID-19 presenting as acute pancreatitis. Pancreatology.

[25] Wang F., Wang H., Fan J., Zhang Y., Wang H., Zhao Q. (2020). Pancreatic Injury Patterns in Patients With Coronavirus Disease 19 Pneumonia. Gastroenterology.

[26] Hayden M.R., Sowers J.R. (2007). Redox Imbalance in Diabetes. Antioxid. Redox Signal..

[27] Pina A.F., Patarrão R.S., Ribeiro R.T., Penha-Gonçalves C., Raposo J.F., Gardete-Correia L., Duarte R., Boavida J.M., Medina J.L., Henriques R. (2020). Metabolic Footprint, Towards Understanding Type 2 Diabetes Beyond Glycemia. J. Clin. Med..

[28] Ahlqvist E., Storm P., Käräjämäki A., Martinell M., Dorkhan M., Carlsson A., Vikman P., Prasad R.B., Aly D.M., Almgren P. (2018). Novel subgroups of adult-onset diabetes and their association with outcomes: A data-driven cluster analysis of six variables. Lancet Diabetes Endocrinol..

[29] Tuomi T., Santoro N., Caprio S., Cai M., Weng J., Groop L. (2014). The many faces of diabetes: A disease with increasing heterogeneity. Lancet.

[30] Zhang S., Wei M., Yue M., Wang P., Yin X., Wang L., Yang X., Liu H. (2018). Hyperinsulinemia precedes insulin resistance in offspring rats exposed to angiotensin II type 1 autoantibody in utero. Endocrine.

[31] Ghadieh H.E., Russo L., Muturi H.T., Ghanem S.S., Manaserh I.H., Noh H.L., Suk S., Kim J.K., Hill J.W., Najjar S.M. (2019). Hyperinsulinemia drives hepatic insulin resistance in male mice with liver-specific Ceacam1 deletion independently of lipolysis. Metabolism.

[32] Najjar S.M., Perdomo G. (2019). Hepatic Insulin Clearance: Mechanism and Physiology. Physiology.

[33] Williamson J.R., Chang K., Frangos M., Hasan K.S., Ido Y., Kawamura T., Nyengaard J.R., Enden M.V.D., Kilo C., Tilton R.G. (1993). Hyperglycemic Pseudohypoxia and Diabetic Complications. Diabetes.

[34] Williamson J.R., Kilo C., Ido Y. (1999). The role of cytosolic reductive stress in oxidant formation and diabetic complications. Diabetes Res. Clin. Pr..

[35] Yan L.-J. (2014). Pathogenesis of Chronic Hyperglycemia: From Reductive Stress to Oxidative Stress. J. Diabetes Res..

[36] Delgado-Roche L., Mesta F. (2020). Oxidative Stress as Key Player in Severe Acute Respiratory Syndrome Coronavirus (SARS-CoV) Infection. Arch. Med. Res..

[37] Weidinger A., Kozlov A.V. (2015). Biological Activities of Reactive Oxygen and Nitrogen Species: Oxidative Stress versus Signal Transduction. Biomolecules.

[38] Cron R.Q., Behrens E.M. (2019). Cytokine Storm Syndrome.

[39] Hotamisligil G.S., Shargill N.S., Spiegelman B.M. (1993). Adipose expression of tumor necrosis factor-alpha: Direct role in obesity-linked insulin resistance. Science.

[40] Duncan B.B., Schmidt M.I., Pankow J.S., Ballantyne C.M., Couper D., Vigo A., Hoogeveen R., Folsom A.R., Heiss G. (2003). Low-Grade Systemic Inflammation and the Development of Type 2 Diabetes: The Atherosclerosis Risk in Communities Study. Diabetes.

[41] Donath M.Y., Böni-Schnetzler M., Ellingsgaard H., Ehses J.A. (2009). Islet Inflammation Impairs the Pancreatic β-Cell in Type 2 Diabetes. Physiology.

[42] Tsalamandris S., Antonopoulos A.S., Oikonomou E., Papamikroulis G.-A., Vogiatzi G., Papaioannou S., Deftereos S., Tousoulis D. (2019). The Role of Inflammation in Diabetes: Current Concepts and Future Perspectives. Eur. Cardiol. Rev..

[43] Böni-Schnetzler M., Meier D.T. (2019). Islet inflammation in type 2 diabetes. Semin. Immunopathol..

[44] Hayden M.R. (2019). Empagliflozin ameliorates tunica adiposa expansion and vascular stiffening of the descending aorta in female db/db mice: An ultrastructure study. Adipobiology.

[45] Catanzaro M., Fagiani F., Racchi M., Corsini E., Govoni S., Lanni C. (2020). Immune response in COVID-19: Addressing a pharmacological challenge by targeting pathways triggered by SARS-CoV-2. Signal Transduct. Target. Ther..

[46] Khaper N., Bryan S., Dhingra S., Singal R., Bajaj A., Pathak C.M., Singal P.K. (2010). Targeting the Vicious Inflammation–Oxidative Stress Cycle for the Management of Heart Failure. Antioxid. Redox Signal..

[47] Ye Q., Wang B., Mao J. (2020). The pathogenesis and treatment of the `Cytokine Storm’ in COVID-19. J. Infect..

[48] Hayden M.R., Patel K., Habibi J., Gupta D., Tekwani S.S., Whaley-Connell A., Sowers J.R. (2008). Attenuation of endocrine-exocrine pancreatic communication in type 2 diabetes: Pancreatic extracellular matrix ultrastructural abnormalities. J. Cardiometabolic Syndr..

[49] Kim J.-W. (2008). Loss of beta-cells with fibrotic islet destruction in type 2 diabetes mellitus. Front. Biosci..

[50] Habibi J., Whaley-Connell A., Hayden M.R., Demarco V.G., Schneider R., Sowers S.D., Karuparthi P., Ferrario C.M., Sowers J.R. (2008). Renin Inhibition Attenuates Insulin Resistance, Oxidative Stress, and Pancreatic Remodeling in the Transgenic Ren2 Rat. Endocrinology.

[51] Yang Y., Kim J.-W., Park H.-S., Lee E.-Y., Yoon K.-H. (2020). Pancreatic stellate cells in the islets as a novel target to preserve the pancreatic β-cell mass and function. J. Diabetes Investig..

[52] Hayden M.R., Yang Y., Habibi J., Bagree S.V., Sowers J.R. (2010). Pericytopathy: Oxidative Stress and Impaired Cellular Longevity in the Pancreas and Skeletal Muscle in Metabolic Syndrome and Type 2 Diabetes. Oxidative Med. Cell. Longev..

[53] Hayden M.R., Sowers J.R. (2011). Childhood-Adolescent Obesity in the Cardiorenal Syndrome: Lessons from Animal Models. Cardiorenal Med..

[54] Hayden M.R. (2007). Islet amyloid and fibrosis in the cardiometabolic syndrome and type 2 diabetes mellitus. J. Cardiometabolic Syndr..

[55] Opie E.L. (1901). The relation of diabetes mellitus to lesions of the pancreas: Hyaline degeneration of the islands of Langerhans. J. Exp. Med..

[56] Hayden M.R., Tyagi S.C. (2001). “A” is for amylin and amyloid in type 2 diabetes mellitus. JOP J. Pancreas.

[57] Hayden M.R., Tyagi S.C. (2000). Remodeling of the endocrine pancreas: The central role of amylin and insulin resistance. South. Med. J..

[58] Jaikaran E.T., Clark A. (2001). Islet amyloid and type 2 diabetes: From molecular misfolding to islet pathophysiology. Biochim. Biophys. Acta (BBA) Mol. Basis Dis..

[59] Hayden M.R., Tyagi S.C. (2002). Islet redox stress: The manifold toxicities of insulin resistance, metabolic syndrome and amylin derived islet amyloid in type 2 diabetes mellitus. JOP J. Pancreas.

[60] Hayden M.R., Tyagi S.C., Kerklo M.M., Nicolls M.R. (2005). Type 2 diabetes mellitus as a conformational disease. JOP J. Pancreas.

[61] Westwell-Roper C.Y., Chehroudi C.A., Denroche H.C., Courtade J.A., Ehses J.A., Verchere C.B. (2014). IL-1 mediates amyloid-associated islet dysfunction and inflammation in human islet amyloid polypeptide transgenic mice. Diabetologia.

[62] D’Alessio D.A., Verchere C.B., Kahn S.E., Hoagland V., Baskin D.G., Palmiter R.D., Ensinck J.W. (1994). Pancreatic Expression and Secretion of Human Islet Amyloid Polypeptide in a Transgenic Mouse. Diabetes.

[63] Haataja L., Gurlo T., Huang C.J., Butler P.C. (2008). Islet Amyloid in Type 2 Diabetes, and the Toxic Oligomer Hypothesis. Endocr. Rev..

[64] Butler P.C., Jang J., Gurlo T., Carty M.D., Soeller W.C., Butler P.C. (2004). Diabetes Due to a Progressive Defect in Cell Mass in Rats Transgenic for Human Islet Amyloid Polypeptide (HIP Rat): A New Model for Type 2 Diabetes. Diabetes.

[65] Hayden M.R., Karuparthi P.R., Manrique C.M., Lastra G., Habibi J., Sowers J.R. (2007). Longitudinal ultrastructure study of islet amyloid in the HIP rat model of type 2 diabetes mellitus. Exp. Biol. Med..

[66] Hayden M.R., Karuparthi P.R., Habibi J., Lastra G., Patel K., Wasekar C., Manrique C.M., Ozerdem U., Stas S., Sowers J.R. (2008). Ultrastructure of islet microcirculation, pericytes and the islet exocrine interface in the HIP rat model of diabetes. Exp. Biol. Med..

[67] Shah A. (2020). Novel Coronavirus-Induced NLRP3 Inflammasome Activation: A Potential Drug Target in the Treatment of COVID-19. Front. Immunol..

[68] Rivera J.F., Gurlo T., Daval M., Huang C.J., Matveyenko A.V., Butler P.C., Costes S. (2010). Human-IAPP disrupts the autophagy/lysosomal pathway in pancreatic β-cells: Protective role of p62-positive cytoplasmic inclusions. Cell Death Differ..

[69] Raleigh D., Zhang X., Hastoy B., Clark A. (2017). The β-cell assassin: IAPP cytotoxicity. J. Mol. Endocrinol..

[70] Coate K.C., Cha J., Shrestha S., Wang W., Fasolino M., Morgan A., Dai C., Saunders D.C., Aramandla R., Jenkins R. (2020). SARS-CoV-2 Cell Entry Factors ACE2 and TMPRSS2 are Expressed in the Pancreas but Not in Islet Endocrine Cells. bioRxiv.

[71] Kusmartseva I., Wu W., Syed F., Van Der Heide V., Jorgensen M., Joseph P., Tang X., Candelario-Jalil E., Yang C., Nick H. (2020). ACE2 and SARS-CoV-2 Expression in the Normal and COVID-19 Pancreas. bioRxiv.

[72] Yang L., Han Y., Nilsson-Payant B.E., Gupta V., Wang P., Duan X., Tang X., Zhu J., Zhao Z., Jaffré F. (2020). A Human Pluripotent Stem Cell-based Platform to Study SARS-CoV-2 Tropism and Model Virus Infection in Human Cells and Organoids. Cell Stem Cell.

[73] Fignani D., Licata G., Brusco N., Nigi L., Grieco G.E., Marselli L., Overbergh L., Gysemans C., Colli M.L., Marchetti P. (2020). SARS-CoV-2 receptor Angiotensin I-Converting Enzyme type 2 is expressed in human pancreatic islet β-cells and in pancreas microvasculature. bioRxiv.

[74] Hudish L.I., Reusch J.E., Sussel L. (2019). β Cell dysfunction during progression of metabolic syndrome to type 2 diabetes. J. Clin. Investig..

[75] Tangvarasittichai S. (2015). Oxidative stress, insulin resistance, dyslipidemia and type 2 diabetes mellitus. World J. Diabetes.

[76] Keane K.N., Cruzat V.F., Carlessi R., de Bittencourt P.I.H., Newsholme P. (2015). Molecular Events Linking Oxidative Stress and Inflammation to Insulin Resistance andβ-Cell Dysfunction. Oxidative Med. Cell. Longev..

[77] Walker N.I., Harmon B.V., Gobé G., Kerr J.F. (1988). Patterns of cell death. Methods Achiev. Exp. Pathol..

[78] Lastra G., Manrique C.M., Hayden M.R. (2006). The Role of Beta-Cell Dysfunction in the Cardiometabolic Syndrome. J. Cardiometabolic Syndr..

[79] Kaiser N., Leibowitz G., Nesher R. (2003). Glucotoxicity and Beta-Cell Failure in Type 2 Diabetes Mellitus. J. Pediatr. Endocrinol. Metab..

[80] Wang S., Ma P., Zhang S., Song S., Wang Z., Ma Y., Xu J., Wu F., Duan L., Yin Z. (2020). Fasting blood glucose at admission is an independent predictor for 28-day mortality in patients with COVID-19 without previous diagnosis of diabetes: A multi-centre retrospective study. Diabetologia.

[81] Wang B., Zhang X., Liu M., Li Y., Zhang J., Li A., Zhang H., Xiu R. (2020). Insulin protects against type 1 diabetes mellitus-induced ultrastructural abnormalities of pancreatic islet microcirculation. Microscopy.

[82] Tomita T. (2016). Apoptosis in pancreatic β-islet cells in Type 2 diabetes. Bosn. J. Basic Med. Sci..

[83] Fonseca S.G., Gromada J., Urano F. (2011). Endoplasmic reticulum stress and pancreatic β-cell death. Trends Endocrinol. Metab..

[84] Hill M.A., Mantzoros C., Sowers J.R. (2020). Commentary: COVID-19 in patients with diabetes. Metabolism.

[85] Singh A.K., Gillies C., Singh R., Singh A., Chudasama Y., Coles B., Seidu S., Zaccardi F., Davies M.J., Khunti K. (2020). Prevalence of co-morbidities and their association with mortality in patients with COVID-19: A systematic review and meta-analysis. Diabetes Obes. Metab..

[86] Ford E.S., Li C., Sattar N. (2008). Metabolic Syndrome and Incident Diabetes: Current state of the evidence. Diabetes Care.

[87] Unanue E.R., Wan X. (2019). The Immunoreactive Platform of the Pancreatic Islets Influences the Development of Autoreactivity. Diabetes.

[88] Wali J.A., Masters S.L., Thomas H.E. (2013). Linking Metabolic Abnormalities to Apoptotic Pathways in Beta Cells in Type 2 Diabetes. Cells.

[89] Lippi G., Lavie C.J., Henry B.M., Sanchis-Gomar F. (2020). Do genetic polymorphisms in angiotensin converting enzyme 2 (ACE2) gene play a role in coronavirus disease 2019 (COVID-19)?. Clin. Chem. Lab. Med..

